# Effect of Anesthesia/Surgery on Gut Microbiota and Fecal Metabolites and Their Relationship With Cognitive Dysfunction

**DOI:** 10.3389/fnsys.2021.655695

**Published:** 2021-08-17

**Authors:** Xinrong Lian, Qianmei Zhu, Li Sun, Yaozhong Cheng

**Affiliations:** ^1^Department of Anesthesiology, National Cancer Center/National Clinical Research Center for Cancer/Cancer Hospital, Chinese Academy of Medical Sciences and Peking Union Medical College, Beijing, China; ^2^National Cancer Center/National Clinical Research Center for Cancer/Cancer Hospital & Shenzhen Hospital, Chinese Academy of Medical Sciences and Peking Union Medical College, Shenzhen, China

**Keywords:** gut microbiota, metabolomics, gut-brain axis, post-operative cognitive dysfunction, neurotransmitter

## Abstract

**Aims:** Post-operative cognitive dysfunction (POCD) is the decline in cognitive function of the central nervous system (CNS) after anesthesia/surgery. The present study explored whether anesthesia/surgery altered gut microbiota and fecal metabolites, examining their associations with risk factors of cognitive dysfunction in aged mice.

**Methods:** Sixteen-month-old C57BL/6 mice underwent abdominal surgery under isoflurane anesthesia to establish an animal model of POCD. The Morris water maze test (MWMT) was used as an indicator of memory after surgery. The effects of anesthesia/surgical interventions on gut microbiota, fecal metabolites, hippocampus, and serum levels of inflammatory factors were examined.

**Results:** The anesthesia/surgery induced more serious POCD behavior, increasing brain interleukin (IL)-6, and IL-1β levels than sham control mice. The relative abundance of bacterial genera *Bacteroidales_unclassified, Mucispirillum*, and *Clostridiales_unclassified* declined, whereas that of *Escherichia*–*Shigella, actinomyces, Ruminococcus_gnavus_group*, and *Lachnospiraceae_FCS020_group* were enriched after anesthesia/surgery compared to the baseline controls. Liquid chromatography–mass spectrometry (LC–MS) showed that the metabolites differed between post-anesthesia+surgery (post_A + S) and baseline samples and were associated with the fecal metabolism of tryptophan, kynurenic acid, N-oleoyl γ-aminobutyric acid (GABA), 2-indolecarboxylic acid, and glutamic acid. Furthermore, the differential metabolites were associated with alterations in the abundance of specific bacteria. These results indicate that the POCD intervention may be achieved by targeting specific bacteria associated with neurotransmitter metabolism.

**Conclusions:** A transient cognitive disturbance induced by anesthesia/surgery may be associated with unfavorable alterations in gut microbiota and fecal metabolites, thereby contributing to the POCD development.

## Introduction

Post-operative cognitive dysfunction (POCD) is the progressive decline in patient cognition and intelligence after anesthesia/surgery. It is clinically characterized by a wide spectrum of symptoms from social withdrawal, lowered concentration to severe cognitive dysfunction. Old age has also been considered as a risk factor for POCD (Luo et al., 2019). As POCD is associated with higher perioperative mortality and inflated medical expenditure for both the patient and society (Steinmetz et al., 2009), it is necessary to clarify the underlying pathophysiological mechanisms of POCD.

Human gut microbiota are a complex microbial community that is potentially vital for human health (Mohajeri et al., 2018). Earlier studies linking alterations in the gut microbiome with neurobehavioral phenotypes have established a conceptual framework of the microbiota–gut-brain axis, whereby gut microbes may influence brain and behaviors through immune, neuronal, and metabolic pathways (Collins et al., 2012; Lynch and Hsiao, 2019). Increasing evidence has suggested that certain microbial members possess the capacity to synthesize and regulate a variety of neurochemicals to control neurotransmission and a mass milieu of other metabolic molecules that indirectly or directly impact neuronal activities (Bauer et al., 2016; Rogers et al., 2016). Gut microorganisms generate bioactive peptides through the transformation of secondary bile acids, short-chain fatty acids, and gut hormones. Short-chain fatty acids, including butyrate, acetate, lactate, and propionate, enter the circulatory system and exert their effects on cellular systems and mediate interactions with gut–brain signaling pathways through endocrine, immune, and neural approaches (Dalile et al., 2019). Microbes can also modulate the metabolism of tryptophan, which performs a critical role in the correct functioning of the immune system and the brain–gut axis (Agus et al., 2018). Three major metabolic pathways of tryptophan that produce kynurenine, indole, and serotonin derivatives are under the indirect or direct control of the microbiota. Dopamine, acetylcholine, and γ-aminobutyric acid (GABA) can also be produced by gut microorganisms (Clarke et al., 2014; Sharon et al., 2019; Dan et al., 2020).

A recent systematic review implicated that gut microbiome function is a key driver of individual variations in functional and cognitive behaviors (Davidson et al., 2018). Previous studies have only surmised that altered gut microbiota are potentially associated with the POCD pathogenesis in aged mice (Jiang et al., 2019; Zhan et al., 2019; Liufu et al., 2020). Due to the significance of microbiota metabolites, researchers have attempted to investigate metabolites, specifically involved in associations with gut microbiota and neurochemicals during disease development. In this study, 16S rRNA sequencing and liquid chromatography–mass spectrometry (LC–MS)-based metabolomic profiling was applied to examine the mechanistic relationship between POCD development and gut microbiota and explore putative biomarkers. Herein, we compared the various characteristics of fecal metabolites, gut microbiota, and inflammatory biomarkers for post-anesthesia/surgery interventions.

## Materials and Methods

### Animals

A total of 50 16-month-old male C57BL/6J mice were supplied by Zhishan Healthcare Research Institute Ltd (Beijing, China). The mice were kept in standard cages under 12 h light/dark cycles at 22.0 ± 2.0°C, with free access to water and food. The mice were randomly divided into a surgery group and a sham control group. The two groups were fed in different cages to prevent the intermixing of gut microbiota. All procedures were performed according to the guidelines of the National Institutes of Health, and the experimental protocol was approved by the Medical Ethics Committee of the National Cancer Center (NCC2019A193).

### Anesthesia and Surgery

Mice were randomly assigned to the anesthesia/surgery group or sham control group by weight. In brief, the mice in the anesthesia/surgery group underwent simple laparotomy under isoflurane anesthesia as previously reported (Ren et al., 2015; Yang et al., 2017). Specifically, anesthesia was induced in each anesthesia/surgery group mouse and maintained with 1.4% isoflurane in 100% oxygen in a transparent acrylic chamber. Fifteen minutes after the induction, the mouse was moved out of the chamber, and isoflurane anesthesia was maintained *via* a cone device. One 16-G needle was inserted into the cone near the nose of the mouse to monitor the concentration of isoflurane. A longitudinal midline incision was made from the xiphoid to the 0.5 cm proximal pubic symphysis on the skin, abdominal muscles, and peritoneum. Then, the incision was sutured layer by layer with 5–0 Vicryl thread. At the end of the procedure, lidocaine cream was administered to the incision site to relieve pain associated with the incision until euthanasia. The procedure for each mouse usually lasted about 15 min. After the surgery plus isoflurane anesthesia, the mice were put back into an anesthesia chamber for up to 2 h of continuous anesthesia with 1.4% isoflurane in 100% oxygen. The rectal temperature of all the mice was maintained at 37 ± 0.5°C using a heating blanket. The mice were put back into their cages and allowed free access to water and food after anesthetic recovery. The mice in the sham control group were placed for 2 h in a similar transparent acrylic chamber with 100% oxygen.

### Behavioral Tests

Spatial learning and memory function were evaluated using the Morris water maze test (MWMT) as previously reported (Vorhees and Williams, 2006). The MWMT was performed in a large circular pool made of white plastic (diameter: 120 cm; height: 50 cm). Water was filled up to a depth of 35 cm at a temperature of 22.0 ± 1.0°C. A 10-cm diameter transparent circular platform was submerged about 0.5–1.0 cm below the water surface in a target quadrant and the pool was placed in a room with diffuse lighting and several wall-mounted visual cues. The swimming activity of the mice was observed and traced using an overhead-mounted camera that relayed data, including total distance traveled, latency to the platform, and the time and distance spent within each quadrant, which was fed into an EthoVision14 tracking system (Noldus Information Technology, Wageningen, The Netherlands). Each mouse was subjected to four trials a day for 5 days in one of the four randomly selected quadrants. Mice were allowed to swim for 60 s to locate the hidden platform, then rest for 30 s on the platform when successful (timed manually using an automatic system). If the platform location failed, the mice were coached to the platform and allowed to remain there for 30 s before being taken back to their cages. On the 6th day, a 60-s probe trial was recorded to evaluate reference memory after the platform was removed. The number of platform-crossing times and time spent in the target quadrant were analyzed.

### Fecal Sample Collection

**Figure 1:**
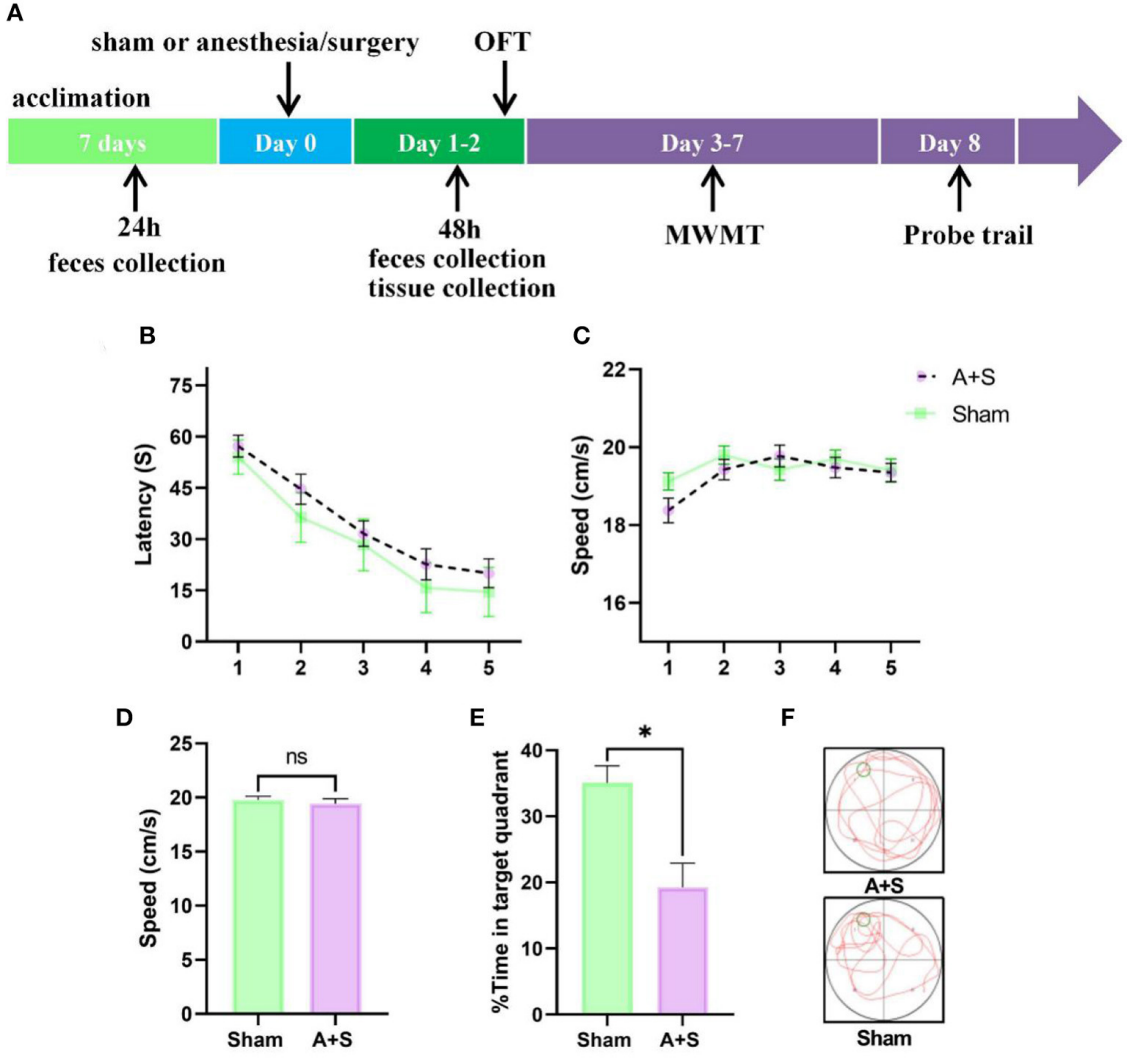
Anesthesia/surgery influences neurobehaviors in aged mice. **(A)** Experimental timeline of the Morris water maze test. **(B)** Escape latency to reach the hidden platform during the 5 days of training (two-way ANOVA, *p* < 0.05). **(C)** Swimming speed during hidden platform training (two-way ANOVA, *p* > 0.05). **(D)** Difference in swimming speed between the two groups during the probe test (unpaired *t*-test, *p* > 0.05). **(E,F)** Time spent in the target quadrant during the probe test (unpaired *t*-test, *p < 0.05). A + S: anesthesia/surgery. *p < 0.05 (*n* = 10 for both sham and A + S groups).

The fecal samples were divided into three types: baseline samples (*n* = 10), the fecal samples collected at 24-h pre-operation from the anesthesia/surgery group; post-anesthesia+surgery (post_A + S) samples (*n* = 10), fecal samples collected 48 h after anesthesia and surgery from the anesthesia/surgery group; and sham group (*n* = 10), fecal samples collected 48 h after anesthesia from the control group (Figure 1A). The samples were put into 1.5-ml tubes, snap-frozen on dry ice, and stored at −80°C. The subsequent steps were conducted for further metabolic profiling and microbial community analysis by LC-Bio Technology Inc (Hangzhou, China).

### 16S rRNA of Fecal Samples

DNA was extracted using a Genomic DNA Kit (Qiagen, Hilden, Germany) as per the protocol of the manufacturer. Bacterial 16S rRNA gene sequences (V3–V4 regions) were amplified from the whole genome using the primers, 341F and 805R. Amplicon libraries were sequenced on an Illumina NovaSeq platform (LC-Bio Technology Co., Ltd., Hang Zhou, Zhejiang Province, China). The raw paired reads of each sample were trimmed by cutting the primer and barcode sequences. Fqtrim v0.94 (https://ccb.jhu.edu/software/fqtrim/index.shtml) was used to filter raw tags for harvesting high-quality clean tags and the Vsearch software (VSEARCH v2.3.4, GitHub, https://github.com/torognes/vsearch) was applied to filter the chimeric sequences. After de-replication, DADA2 was used to obtaining the sequences and feature table. Alpha and beta diversities were evaluated by applying random normalization to the same sequences, then based upon the SILVA classifier v132 (from Latin silva, forest, http://www.arb-silva.de), feature abundance was normalized to the relative abundance of each sample. Alpha diversity was inferred by the number of observed species and the indices of Chao (species richness) and Shannon (diversity). Beta diversity was calculated by QIIME2 (https://pubmed.ncbi.nlm.nih.gov/31399723/). Principal coordinate analysis (PCoA) plots were drawn based on the unweighted and weighted distance metrics of UniFrac, and principal component analysis (PCA) was performed to compare gut microbiota composition before and after intervention between the different groups at phylum and genus levels.

### Fecal Metabolic Analysis and Data Processing

The metabolites were extracted from fecal samples using a 50% methanol buffer and incubated for 10 min at 24°C. The extracted mixture was centrifuged for 20 min at 4,000 × *g*, and the supernatants were analyzed using LC–MS to identify the metabolites. Chromatographic separations were performed using an ultra-performance liquid chromatography (UPLC) system (SCIEX, Framingham, USA, https://www.sciex.com/) and ACQUITY UPLC HSS T3 column (Waters, Elstree, UK). A high-resolution tandem mass spectrometer, TripleTOF 5600 Plus (SCIEX, UK), was operated in both PIM and NIM to detect metabolites eluted from the column. The mass range of TOF was from 60 to 1,200 Da. The XCMS software was used for the acquired MS data pretreatments. The LC-MS raw data files were processed by metaX that used the package XCMS for peak detection and CAMERA package for peak annotation, all based on R software (R, Vienna, Austria, http://www.R-project.org/). Each ion was identified by combining retention time and *m*/*z* data. Orthogonal projection to latent structure discriminant analysis (OPLS-DA) was performed using the SIMCA-P software package (v14.1) (Umetrics, Umea, Sweden) to examine the overall distribution of microbial metabolites between the two groups at baseline and after the intervention. The Kyoto Encyclopedia of Genes and Genomes (KEGG) database was to annotate the metabolites. A *p-*value (*t*-test) of <0.05 and variable importance in projection (VIP) of ≥1 was used to identify significant metabolites. Metabolites with a fold change (FC) of >1.25 were selected for further analysis.

### Association Between Metabolites and Gut Microbiota

Spearman's correlation was calculated to determine the association between differential metabolites and gut microbiota due to anesthesia/surgery using the Hmisc package in R (v3.4.3, R, Vienna, Austria, http://www.R-project.org/). The top 38 species of the two groups and metabolites with more than 2-FC between post_A + S and baseline, VIP of ≥ 1, and *p* < 0.05 were identified.

### Evaluation of Inflammatory Factors in Brain and Serum Samples

Mouse serum and hippocampus were harvested at 48-h post-operation. Levels of serum or hippocampus inflammatory factors, such as interleukin (IL)-1β, tumor necrosis factor (TNF)-α, and IL-6, were assessed using an ELISA kit (Shanghai Enzyme-linked Biotechnology Inc, Shanghai China) as per the instructions of the manufacturer. All measurements were obtained at the end of the study to minimize variability.

### Statistical Analysis

All values are presented as mean ± SEM. For the behavior tests, the two-way repeated measurements of ANOVA were used to analyze water maze escape latency and average speed. One-way ANOVA was used for the probe quadrant trial data. The Student's *t*-test was used for comparisons between two groups. The online tool, OmicStudio (https://www.omicstudio.cn/tool) was used to perform the Wilcoxon rank-sum test for significantly altered bacterial features. A value of *p* <0.05 was regarded as statistically significant. All statistical analyses were performed using the GraphPad Prism 8.3.0 (GraphPad Software, San Diego, California, USA) and R version 3.4 software (R, Vienna, Austria).

## Results

### Effects of Anesthesia/Surgery on Cognitive Impairment in Mice

To investigate whether anesthesia/surgery induced cognitive dysfunction in aged mice, MWMT was used to evaluate learning and memory after abdominal surgery plus isoflurane anesthesia. Each mouse was permitted to rest for 2 days post-operation. The training course of the water maze was used for 5 days, then probe tests were performed on day 8 (Figure 1A). On the training course, mice from the anesthesia/surgery group showed significantly longer escape latency than the sham group (Figure 1B). No significant intergroup difference in swimming speed was found during the training or probe tests, indicating a post-operational influence of perceptual or motor abilities on memory and spatial learning (Figures 1C,D). In the probe tests, mice in the sham group learned to use spatial cues to navigate a direct path to the quadrant with a hidden platform and exhibited a significant preference for the target quadrant after training. Nevertheless, this preference for the target quadrant was significantly compromised in operated mice compared to control mice (Figures 1E,F), indicating that anesthesia/surgery damaged reference memory in aged mice.

### Effects of Anesthesia/Surgery on Gut Microbiota

To understand alterations in the gut microbiome during anesthesia/surgery, 16S rRNA sequencing was conducted on fecal samples of the anesthesia/surgery and sham groups. The datasets presented in this study can be found in the online repository, the names were deposited in the European Nucleotide Archive under accession number PRJEB43448. An operational taxonomic unit (OTU) was employed to classify microbial diversity in bacterial strains based upon rRNA sequence similarity. The Venn diagram displayed 1,801 + 881 OTU for the baseline group and 1,199 + 881 OTU for the post_A + S group, whereas 881 OTUs were common between the two groups (Supplementary Figure 1A). There were 2,035 + 807 OTU in the sham group and 624 OTU in all three groups (Supplementary Figures 1B,C).

**Figure 2:**
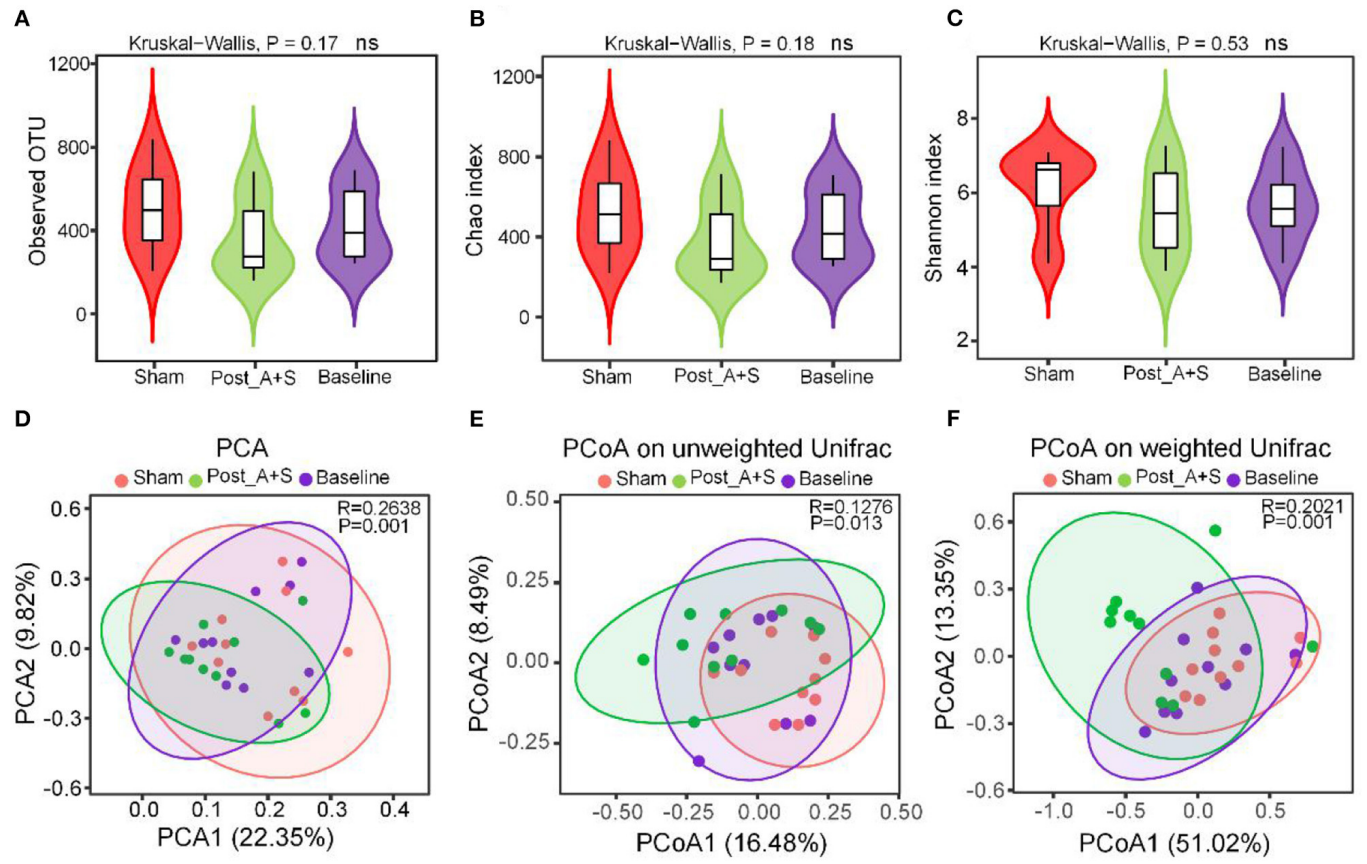
Altered gut microbiota affected by anesthesia/surgery. **(A)** Operational taxonomic units (OTUs) of microbiota in each sample. **(B)** Chao 1 index for each sample. **(C)** Shannon index for each sample. **(D)** Principal component analysis (PCA) of the three types of fecal samples. **(E)** PCoA of the unweighted UniFrac of the three types of fecal samples. **(F)** PCoA of the weighted UniFrac of the three types of fecal samples (*n* = 10 for both the sham, baseline, and A + S groups).

Alpha diversity analysis was used to examine the complexity of species diversity using the observed species, Chao and Shannon indices, with the average OTUs similar among the three groups (Figure 2A). Microbial community richness as indicated by the Chao and Shannon indices were also similar (Figures 2B,C), indicating that the diversity of microbial species was not significantly different between the three groups. The beta diversity analysis is the comparison of diversity between different ecosystems (Nakov et al., 2015). PCA and PCoA demonstrated that the global microbiome composition and abundances were significantly different among the three groups (Figures 2D,F). PCoA plots of UniFrac distance dissimilarity revealed that the sham samples were closest to the baseline samples, whereas the dots of the post_A + S sample were further away from that of the two other samples (Figures 2E,F).

**Figure 3:**
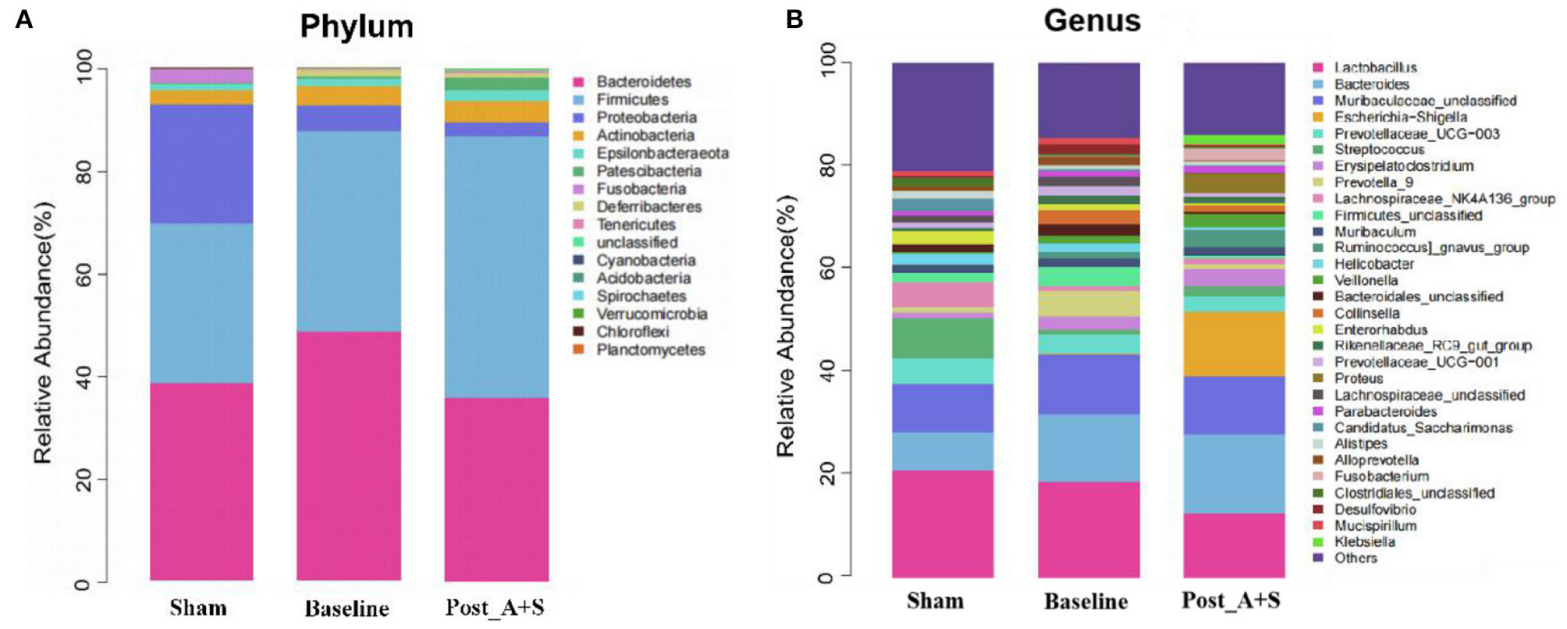
**(A)** Relative abundance of the microbiota at phylum level in the different fecal samples. **(B)** Relative abundance of the microbiota at genus level in the different fecal samples (*n* = 10 for both the sham, baseline, and A + S groups).

The top 20 differential microbial profiles among the three groups displayed similar patterns at both phylum and genus levels (Figures 3A,B). The post_A + S sample showed significant declines of *Deferribacteres*, whereas *Fusobacteria* and *Proteobacteria* at the phylum level were enriched compared to the baseline group after anesthesia/surgery (Figure 3A, Supplementary Figure 2A). At the genus level, the genera *Escherichia*–*Shigella* and *Fusobacterium* increased in abundance in the post_A + S sample, whereas the baseline or sham group samples showed lower abundance levels after anesthesia/surgery (Figure 3B, Supplementary Figures 2A,B). *Escherichia*–*Shigella* positively correlated with Alzheimer's disease, probably related to a peripheral inflammatory state in mice with brain amyloidosis and cognitive dysfunction (Cattaneo et al., 2017). The same findings have been previously reported in APP/PS1 mice (Chen et al., 2020). Interestingly, the genus levels of both *Bacteroidales_unclassified* and *Lachnospiraceae_UCG-001* significantly decreased in the post_A + S sample compared to the baseline sample (Supplementary Figure 2A). Furthermore, post_A + S samples showed different gut microbiota types compared to the sham samples (Supplementary Figure 2B); thus, POCD may be significantly associated with the altered abundance of specific gut bacteria.

### Effects of Anesthesia/Surgery on Fecal Metabolomics Profiles

**Figure 4:**
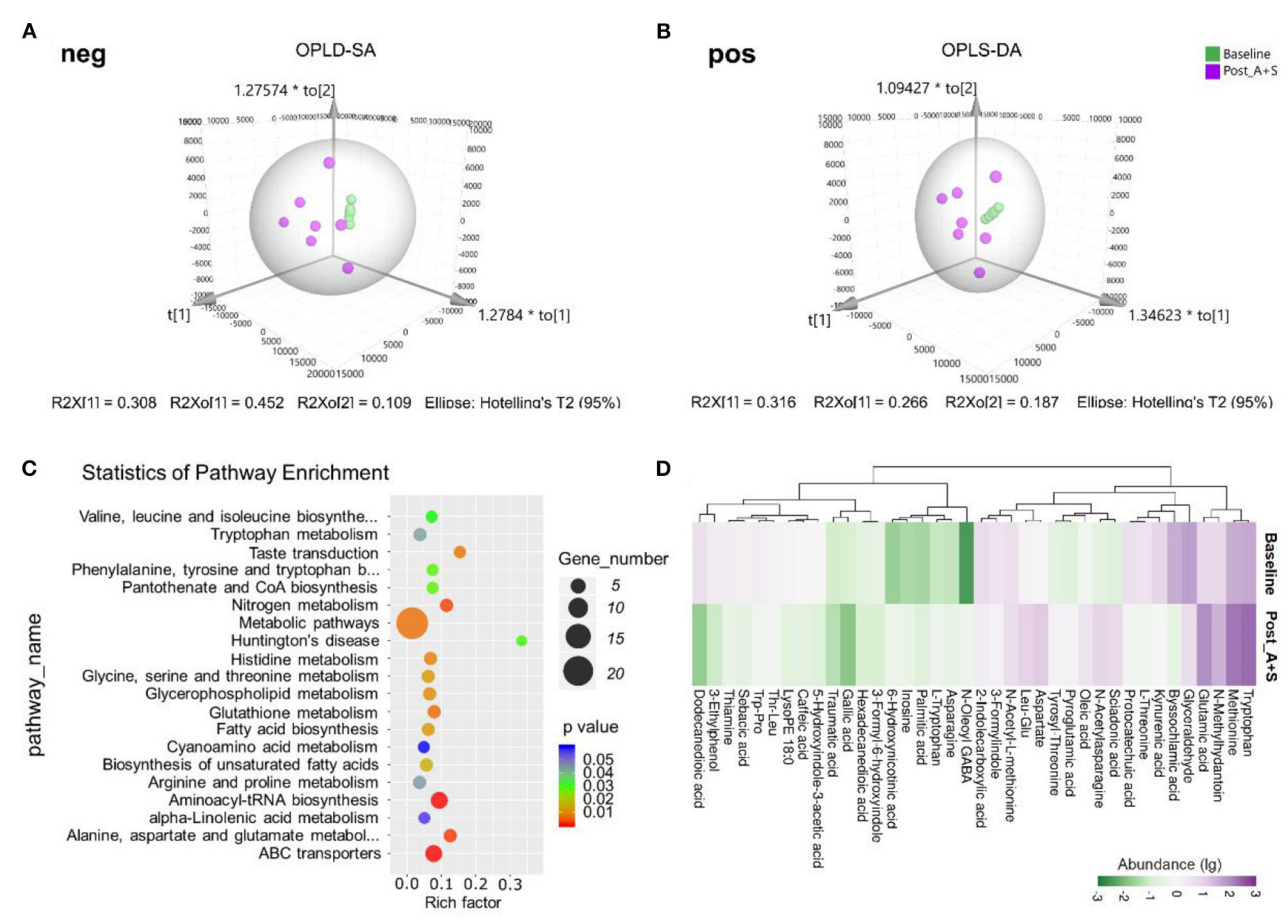
Effects of anesthesia/surgery on fecal metabolomic profiles. **(A)** OPLD-SA in the negative ion mode. **(B)** OPLD-SA in the positive-ion mode. **(C)** The Kyoto Encyclopedia of Genes and Genomes (KEGG) pathway enrichment analysis of the dysregulated metabolites. **(D)** The abundance of metabolites in the post_A + S and baseline samples (*n* = 7 for both baseline and post_A + S groups).

Mounting evidence has demonstrated that certain metabolites of gut microbiota may enter the blood circulation and regulate the behaviors and physiology of hosts (Sarkar et al., 2016; Yang and Duan, 2018; Zalar et al., 2018). Metabolomic analysis yielded 8,232 and 9,560 features in the positive and negative ion modes, respectively (Table 1). The comparative metabolomics analysis determined that the change in fecal metabolites in the post_A + S and baseline samples showed a significant difference in the profile of metabolites (Supplementary Figure 3A), with 1,264 dysregulated (678 upregulated and 586 downregulated) features identified in the Pos mode (Supplementary Figure 3B) and 1,352 dysregulated (500 upregulated and 852 downregulated) metabolites identified in Neg mode (Supplementary Figure 3C). Fecal samples from different groups were classified based on the OPLS-DA (Figures 4A,B), suggesting that anesthesia/surgery led to significant biochemical changes.

**Table 1:** Statistical analysis of the fecal metabolome.

**Mode**	**All features**	**All annotated**	**Comparison**	**Up**	**Down**
Pos	8,232	5,492	post_A + S/baseline	678	586
Neg	9,560	5,358	post_A + S/baseline	500	852

*A + S, anesthesia/surgery*.

Metabolic features were identified and compared using the online databases, Human Metabolome Database, and KEGG. The KEGG pathway enrichment analysis of the dysregulated metabolites revealed a close association between metabolic pathways and the biosynthesis of secondary metabolites after anesthesia/surgery (Figure 4C). The most significantly enriched pathways were correlated with the metabolism of tryptophan, glutathione, histidine, and amino acids (Figure 4C). The tryptophan metabolic pathway, in particular, has been implicated in neuropsychiatric disorders (Jenkins et al., 2016; Gheorghe et al., 2019; Kaluzna-Czaplinska et al., 2019). Tryptophan can be metabolized into endogenous tryptophan metabolites (kynurenine, tryptamine, and indole) and bacterial tryptophan metabolites (indole, indolic acid, skatole, and tryptamine), which modulate neuroendocrine and intestinal immune responses (Gao et al., 2018, 2020).

Among those metabolites matched to the KEGG database, the abundances of 76 metabolites were significantly altered between post_A + S and baseline samples (Supplementary Tables 1, 2). Interestingly, 38 different metabolites with 2-FC were significantly altered in post_A + S samples compared to the baseline sample (Figure 4D). Tryptophan, oleoyl GABA, glutamic acid, and asparagine were more abundant in post_A + S samples.

**Figure 5:**
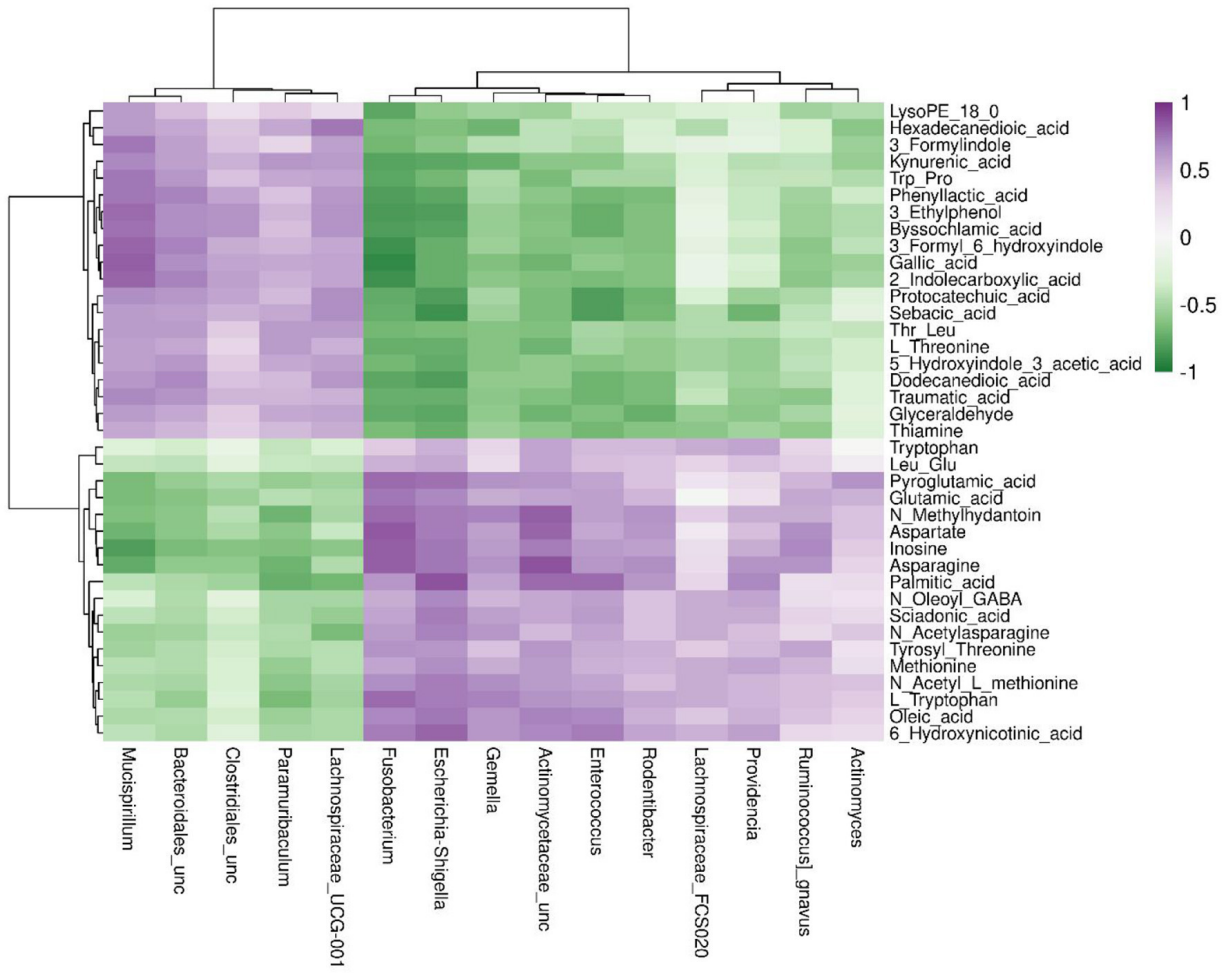
Spearman's correlation analysis of the metabolic products and gut microbiome in the post-A + S and baseline groups. The top 15 genera were detected using 16S rRNA data. Metabolites with fold change of >2 between post-A + S and baseline, *p* < 0.05 (*t*-test), and variable importance in projection (VIP) ≥ 1. The correlation effect is indicated using a color gradient from green (negative correlation) to purple (positive correlation).

Spearman correlation was applied to investigate potential associations between changes in metabolic products and the gut microbiome (Figure 5). The abundance of microbiota belonging to genera, such as *Fusobacterium, Actinomycetaceae_unclassified*, and *Escherichia*–*Shigella*, was positively correlated with levels of L-tryptophan, N-methylhydantoin, aspartate, glutamic acid, and 6-hydroxynicotinic acid. As the only precursor of serotonin, tryptophan functions as a critical monoamine neurotransmitter involved in modulating intestinal physiological function and central nervous transmission. In summary, anesthesia/surgery altered the composition and structure of gut microbiota and substantially changed the composition of fecal metabolites in mice.

### Effects of Anesthesia/Surgery on Inflammatory Markers

Interactions between inflammation and the central nervous system (CNS) have been observed in experimental POCD (Rosczyk et al., 2008; Hovens et al., 2013, 2014; Yang et al., 2018). To determine whether gut microbiota participates in the systemic inflammatory response, the mice were sacrificed at 48 h after anesthesia/surgery, and hippocampus and serum samples were collected. As shown in Table 2, the levels of IL-1β and IL-6 significantly increased in the hippocampus of mice in the A + S group compared to the sham group (*p* < 0.05). However, no significant differences in serum IL-1β, IL-6, and TNF-α were found between the two groups (*p* > 0.05, Table 3).

**Table 2:** Effects of anesthesia/surgery on hippocampus inflammatory markers.

**Inflammatory markers**	**Sham (*n* = 10)**	**A + S (*n* = 10)**	***p-*value**
IL-6 (pg/ml)	13.11 ± 0.56	19.93 ± 0.73	<0.01
IL-1β (pg/ml)	13.24 ± 0.59	18.81 ± 0.61	<0.01

*Data are shown as mean ± SEM (n = 10). p < 0.05 compared with the sham group. A + S: anesthesia/surgery*.

**Table 3:** Effects of anesthesia/surgery on serum inflammatory markers.

**Inflammatory markers**	**Sham (*n* = 10)**	**A + S (*n* = 10)**	***p*-value**
IL-6 (pg/ml)	10.43 ± 0.70	10.52 ± 0.63	0.57
IL-1β (pg/ml)	13.05 ± 1.33	11.86 ± 1.81	0.08
TNF-α (pg/ml)	53.78 ± 4.71	54.77 ± 7.91	0.74

*Data are shown as mean ± SEM (n = 10). p > 0.05 compared with the sham group. A + S: anesthesia/surgery*.

## Discussion

This study determined the effects of the anesthesia/surgery on behaviors, gut microbiota, metabolites, brain IL-6, and IL-1β levels in old mice, showing that the anesthesia/surgery induced greater behavioral changes, increases in brain IL-6 and IL-1β levels, induced gut microbiota phylum and genus levels, and regulated intestinal metabolites in A + S old mice compared to sham old mice.

Recently, many researchers have found associations between gut microbiota composition and cognitive impairment using 16S rRNA sequencing (Cattaneo et al., 2017; Han et al., 2020). As reported in Zhang et al. (2019), gut microbiota composition was different between 8-week old mice with cognitive impairment in post-operative delirium-like behaviors and control old mice; the former had higher levels of gammaproteobacterial, whereas the latter had higher levels of tenericutes and mollicutes. Liufu et al. found that induced by anesthesia/surgery, gut microbiota of the mice and their associated post-operative behaviors were both changed; correspondingly, treating these 18-month-old mice with Lactobacillus or a probiotic could mitigate their post-operative behaviors of cognitive disorder (Liufu et al., 2020). In addition, it was observed in this study that anesthesia/surgery caused the behavioral changes associated with age, dysbiotic microbiota, increased levels of IL-6 and dysfunctional mitochondria in the brain, and also increased levels of synaptic markers among the mice. Jiang et al. also found that impaired cognitive function and alternated gut microbiota could be caused by anesthesia/surgery among those 18-month-old mice, and mitigated by probiotics (Jiang et al., 2019).

In this study, we first described the gut microbiota composition in an animal anesthesia/surgery model. The post_A + S sample showed a different gut microbiota composition compared with baseline and sham samples. The abundance of the phyla *Fusobacteria* and *Proteobacteria* were enriched in the post_A + S sample, which was in relatively poorer health. *Fusobacteria* was highly heterogeneous (Wang and Wang, 2016) and some species, specifically *Fusobacterium nucleatum*, caused an inappropriate inflammatory response in intestinal epithelial cells. *Proteobacteria* are involved in inflammatory and immunological reactions and function as the microbial signature of dysbiosis in various diseases. Moreover, the abundance of the genus *Escherichia*–*Shigella* was significantly elevated in post_A + S, compared to the baseline conditions. It has also been reported to be related to the proinflammatory status since it can produce proinflammatory cytokines *via* an NLRP3-dependent mechanism (Morgan, 2013; De la Fuente et al., 2014). The gut immune system is a potential communication route between gut microbiota and the brain, and the balance of gut microbiota may regulate the inflammatory response, which may also regulate emotions and behavior (Wang and Wang, 2016). Taken together, these alterations at the genus level suggest that anesthesia/surgery exerts a selective effect on gut microbiota. However, further studies are needed to investigate the functions of these bacteria after anesthesia/surgery.

Increasing evidence has demonstrated that several metabolites of the gut microbiota can enter the circulatory system and regulate the physiologies and behaviors of hosts (Collins et al., 2012; Sarkar et al., 2016; Dinan and Cryan, 2017). In this study, fecal metabolic phenotyping was performed on aged mice after anesthesia/surgery. Fifteen differentially expressed metabolites were identified, which are mainly involved in the metabolism of nucleotides, fatty acids, and amino acids, and partially involved in neurotransmitter metabolism.

Tryptophan and its metabolites play important roles in the proper functioning of the immune system and the brain–gut axis (Bosi et al., 2020). 5-HT, a tryptophan metabolite, has been identified as a key developmental regulator of the intestinal system and the CNS, and it may play a role as a nexus for the gut–brain axis in children on the autism spectrum (Chen et al., 2017; Israelyan and Margolis, 2018). Kynurenine, another tryptophan metabolite, is altered in both the periphery system and the CNS of patients with Parkinson's disease, and its changes are correlated with symptom severity (Heilman et al., 2020). As revealed through our analysis, tryptophan levels were significantly elevated after anesthesia/surgery compared to baseline. However, specific regulatory bacteria of tryptophan and tryptophan metabolites remain unknown. Interestingly, the enriched genus *Escherichia*–*Shigella* in the post_A + S sample was positively correlated with tryptophan, suggesting that elevated tryptophan levels might be due to *Escherichia*–*Shigella* enrichment in mice after anesthesia/surgery. Moreover, *Fusobacterium* and *Escherichia*–*Shigella* showed a high correlation with reduced kynurenic acid levels, suggesting that reductions of both genera in the post_A + S sample resulted in metabolic abnormalities of the kynurenine pathway *via* the regulation of amino acid metabolism in POCD.

Numerous research studies have provided the evidence that dopamine is correlated with motivational and cognitive control of behaviors (Cools, 2008). However, a dopamine-related hypothesis that links neurobiology to the POCD behavior has not yet been put forward. The present analysis revealed that certain significantly altered phenylalanine and tyrosine derivatives are associated with the synthesis and metabolism of dopamine. Furthermore, *Fusobacterium* and *Escherichia*–*Shigella* levels were significantly correlated with these differential metabolites, suggesting that genera enriched in the post_A + S sample may potentially induce dopamine signaling abnormalities in POCD by modulating the metabolism of amino acids.

N-oleoyl γ-aminobutyric acid is a major inhibitory neurotransmitter of the CNS (Duman et al., 2019) and has been recently demonstrated to change in children with autism spectrum disorder (ASD) compared to normal children, which may correlate with gut dysbiosis (Kang et al., 2018; Dan et al., 2020). Anesthesia/surgery contributed to neuroinflammation in the hippocampus, thus disrupted the GABAergic system. Surface α5GABAARs are upregulated through the P38 MAPK pathway, finally resulting in a hippocampus-dependent memory defect (Zhang et al., 2020). In agreement with the above findings, the GABA precursors pyroglutamic acid and glutamate acid were enriched after anesthesia/surgery (Strandwitz et al., 2019), implying that elevated levels of GABA precursors might be induced by enriched *Escherichia*–*Shigella*. A disrupted GABAergic system resulted in perioperative neurocognitive disorder development after anesthesia or surgery in advanced-age mice (Zhang et al., 2020). Thus, further research should focus on the specific microbiota related to GABA-receptor-mediated functions in causative regions of POCD.

Neuroinflammation has been considered to be associated with POCD (Terrando et al., 2011). To be specific, IL-6 was related to the impairment of learning and memory among the animals (Braida et al., 2004; Cao et al., 2010), and cognitive dysfunction among the patients (Patanella et al., 2010). Consistent with these findings, it was found in this study that brain IL-6 and IL-1β levels were increased by anesthesia/surgery among the A + S mice (Table 2). However, as shown in Table 3, the levels of IL-1β, IL-6, and TNF-α in serum were found with no significant differences between groups 48 h after anesthesia/surgery. It was speculated that surgery-related inflammatory alterations may be caused by different experimental settings, such as murine models, sampling time points, and tissue location. In addition, pathogens and inflammation mediators could enter into the bloodstream through the “leaky gut,” thereby promoting the interaction between intestinal microbiota alteration and CNS dysfunction (Erny et al., 2015; Alhasson et al., 2017). In other words, intestinal dysbiosis is related to intestinal inflammation and barrier integrity and may affect the CNS via gut–brain axis (Han et al., 2020). The prebiotics intervention effectively attenuated intestinal microbiota alteration and inflammatory responses, thereby reducing surgery-induced cognitive dysfuntion. Moreover, a study indicated that probiotics alleviate cognitive dysfunction associated with neuroinflammation in cardiac surgery (Yu et al., 2019; Han et al., 2020). Based on the established system, we will further explore the changes in the blood levels of these inflammatory cytokines, or the other microbiota inflammatory cytokines, lipopolysaccharides, and TLR2/TLR4 at multiple time points within 24 h after anesthesia/surgery in future studies.

Fecal microbiota transplantation is a treatment to introduce the fecal microbiota obtained from a healthy donor into the colon of a patient, thereby restoring the normal microbial composition of the intestinal tract. Some studies have suggested that fecal microbiota transplantation can improve the symptoms of depression and anxiety. Moreover, increased diversity of intestinal flora may improve mood (Kurokawa et al., 2018). When gut microbiota from human donors with ASD or typically developing (TD) controls were transplanted into germ-free mice, the colonization of ASD microbiota was sufficient to induce ASD-like behaviors (Sharon et al., 2019). When there are differences in microbial composition, fecal microbiota transplantation of specific flora can improve the symptoms in patients with Parkinson's disease (Kang et al., 2017). In addition, when the fecal microbiota from patients with Parkinson's disease was transplanted into the alpha-synuclein overexpressing mice, motor symptoms of these humanized mice could be exacerbated compared to the healthy controls (Sampson et al., 2016). Although the specific bacteria involved in the anesthesia/surgery-induced cognitive decline was not identified in this study, further investigations are needed to address these issues, such as fecal transplants into germ-free mice, intestinal immune steady-state, microglia activation and inflammation, and the effects of microbial metabolites on neural activity.

One major limitation of this study was that the fecal sample was collected only at baseline and 48-h post-operation. Moreover, the mice in both groups lost weight, and the weight loss was different between groups after the surgical intervention. Whether weight loss resulted in the alterations in gut microbiota and fecal metabolomics or vice versa requires further study. Another limitation was related to the universality of the subjects, which were only healthy mice, so that the findings may not be applicable to subjects at a high risk of metabolic disorders. Although there was a potential application of gut microbiota in the pathogenesis of POCD, future studies identifying critical bacteria species may make clear any alterations of metabolites and neuro-inflammation.

## Conclusion

In summary, mice that underwent anesthesia/surgery demonstrated gut dysbiosis at the levels of phylum and genus. The genus associated with altered metabolites were identified in the post_A + S sample. The relationships between metabolites, gut microbiota, and neurotransmitters may offer avenues for elucidating the underlying mechanisms of altered cognitive behaviors in POCD and confirm whether the origin of such alterations is associated with gut microbiota. Aberrant metabolites of fecal microbiota might exert strong pathogenic implications for the gut–brain axis in POCD. The present study shed light on the relationship between metabolites, fecal microbiome, gut dysbiosis, and deregulation of neurotransmitters in POCD mice. Future therapeutic modalities may be developed for the POCD intervention *via* targeting specific microbiota related to neurotransmitter metabolism.

## Data Availability Statement

The datasets presented in this study can be found in online repositories. The names of the repositories and accession number can be found below: 1. European Nucleotide Archive (ENA) and accession number PRJEB43448; 2. National Center for Biotechnology Information (NCBI) SRA and SRP307478 (accession: SRX10148594~SRX10148623).

## Ethics Statement

The animal study was reviewed and approved by Medical Ethics Committee of National Cancer Center (NCC2019A193).

## Author Contributions

XL and QZ implemented the model and analyzed the data. YC provided theoretical analysis and. LS supervised the project. XL wrote the manuscript. All authors contributed to the article and approved the submitted version.

## Conflict of Interest

The authors declare that the research was conducted in the absence of any commercial or financial relationships that could be construed as a potential conflict of interest.

## Publisher's Note

All claims expressed in this article are solely those of the authors and do not necessarily represent those of their affiliated organizations, or those of the publisher, the editors and the reviewers. Any product that may be evaluated in this article, or claim that may be made by its manufacturer, is not guaranteed or endorsed by the publisher.
