# Supplementary material for: Effect of Anesthesia/Surgery on Gut Microbiota and Fecal Metabolites and Their Relationship With Cognitive Dysfunction
Source: Front Syst Neurosci. 2021 Aug 17;15:655695. doi: 10.3389/fnsys.2021.655695 (PMC8416053; doi:10.3389/fnsys.2021.655695)
Supplement: Supplementary file 3 [file Data_Sheet_1.docx]

**
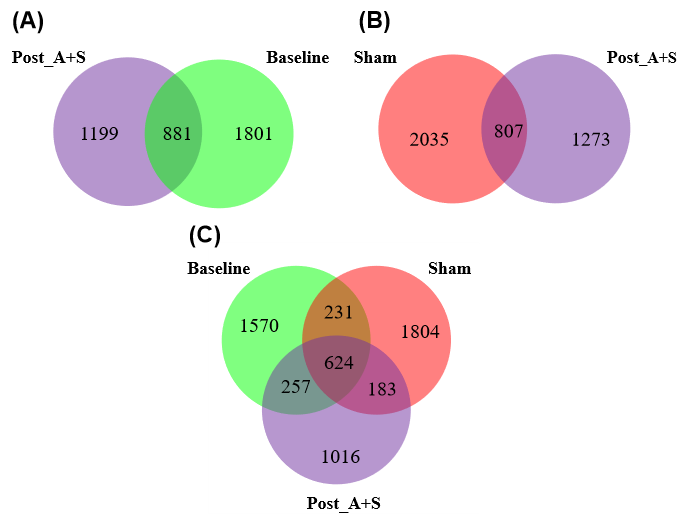
**

**Figure S1.** Venn diagram shows the common OUT among anesthesia/surgery and sham groups. (A) OTU between post_A+S and baseline samples. (B) OTU between Sham and post_A+S samples. (C) OTU among the three types of fecal samples. A+S = anesthesia/surgery.


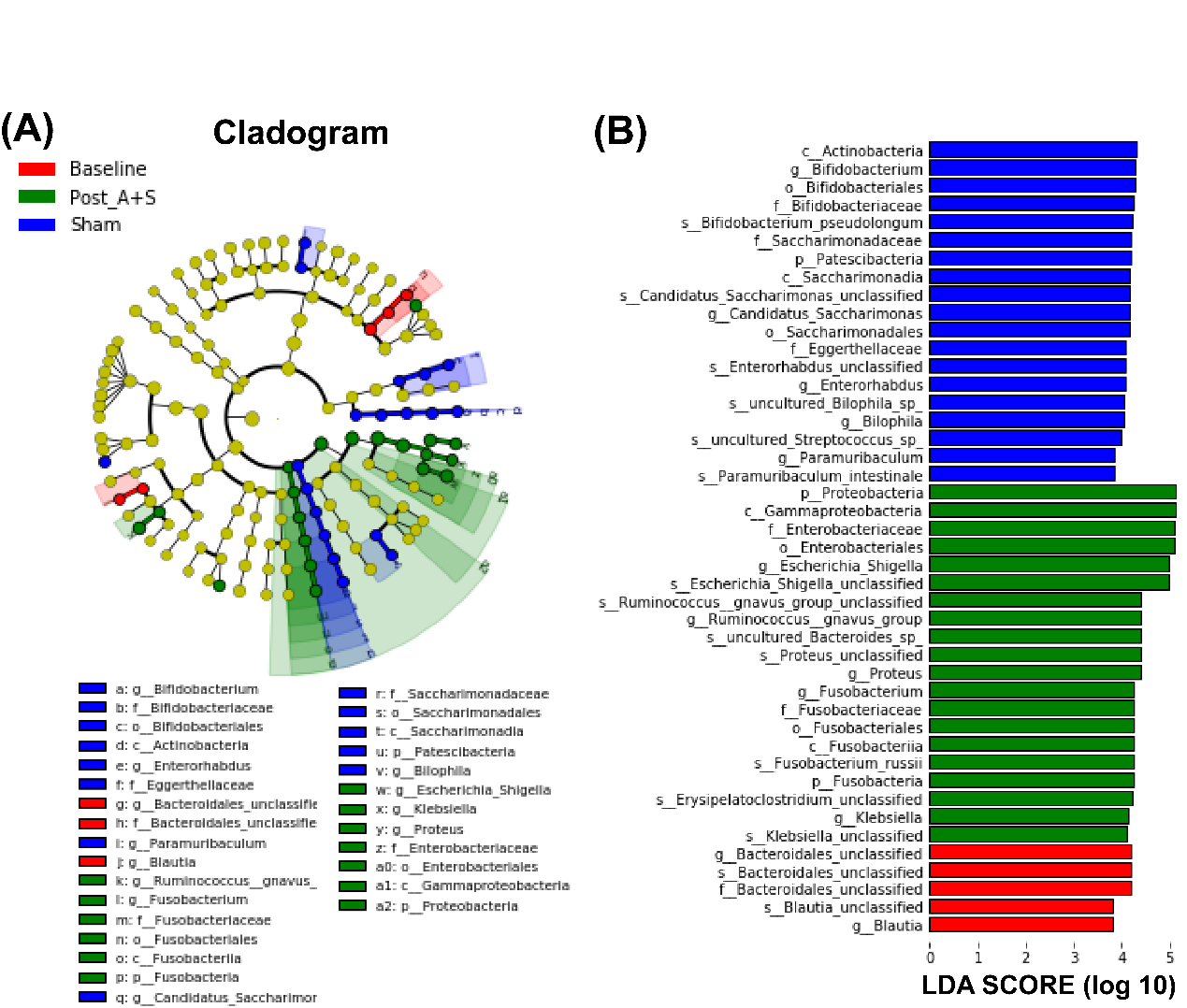


**Figure S2.** Anesthesia/surgery induced intestinal flora dysbiosis. (A) Phylogenetic distribution of fecal microbiota differences among the three groups. Green color is represented with significant differences of taxonomic representation in the post A+S groups, red color represents significant differences in taxonomic representation in the baseline groups, blue color represents significant differences in taxonomic representation in the sham groups. The fecal microbiota shared in three groups is marked by yellow color. (B) The taxonomy of the intestinal flora was analyzed by linear discriminant analysis (LDA) with effect size measurements (LEfSe). The length of bars in the chart represents the significance of differences in fecal microbiota among three groups. For LDA scores >2, *P*<0.05 (n = 10). Compositions of different species of fecal microbiota associated with anesthesia/surgery and sham mice assessed by LEfSe analysis. A+S = anesthesia/surgery.

**(A)**


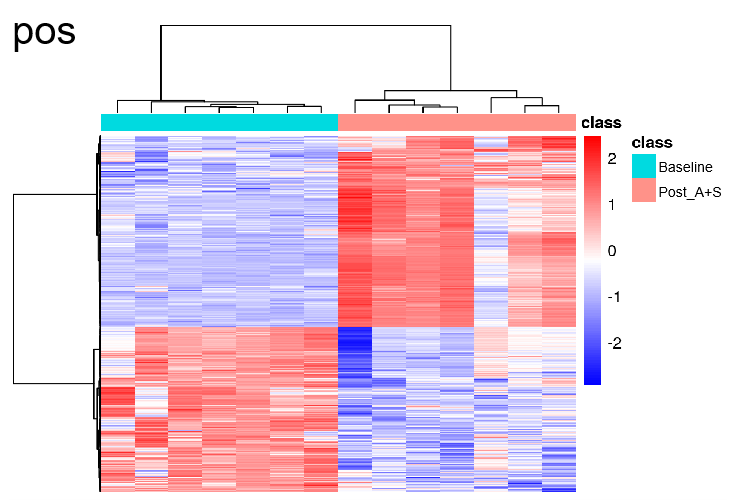

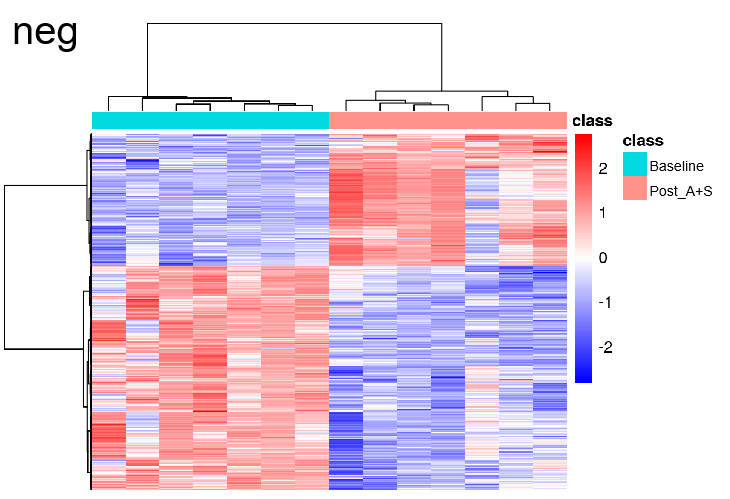


**(B) (C)**


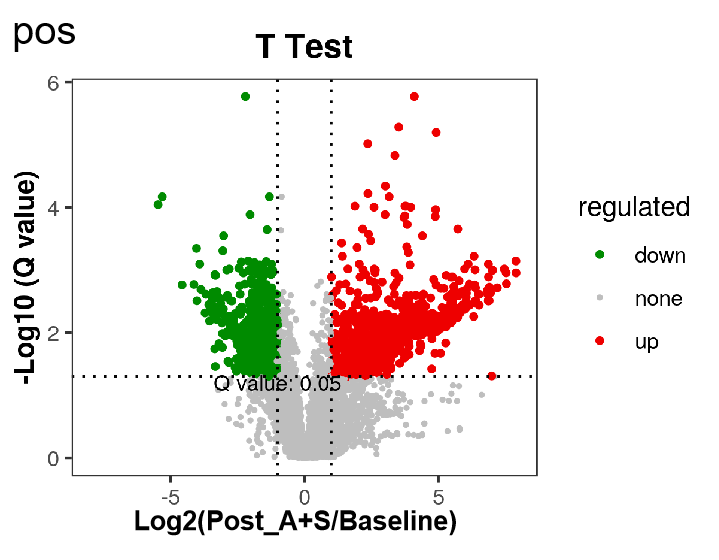

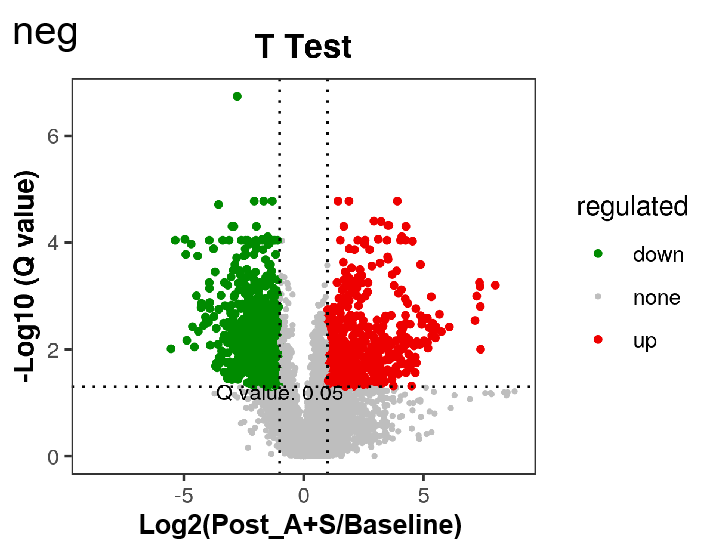


**Figure S3**. (A) Heatmap of dysregulated metabolites between post_A+S and baseline samples in pos and neg mode respectively. (B) Volcano plot of dysregulated metabolites between post_A+S and baseline samples in pos and neg mode respectively (Student’ t test, *P* value < 0.05).
